# The effects of different enrofloxacin dosages on clinical efficacy and resistance development in chickens experimentally infected with *Salmonella* Typhimurium

**DOI:** 10.1038/s41598-017-12294-7

**Published:** 2017-09-15

**Authors:** Jun Li, Haihong Hao, Guyue Cheng, Xu Wang, Saeed Ahmed, Muhammad Abu Bakr Shabbir, Zhenli Liu, Menghong Dai, Zonghui Yuan

**Affiliations:** 1 National Reference Laboratory of Veterinary Drug Residues (HZAU) and MOA Key Laboratory for Detection of Veterinary Drug Residues, Wuhan, China; 2 MOA Laboratory for Risk Assessment of Quality and Safety of Livestock and Poultry Products, Wuhan, China; 3 Hubei Collaborative Innovation Center for Animal Nutrition and Feed Safety, Huazhong Agricultural University, Wuhan, Hubei 430070 China

## Abstract

To investigate the optimal dosage which can improve clinical efficacy and minimize resistance, pharmacokinetics/pharmacodynamics model of enrofloxacin was established. Effect of enrofloxacin treatments on clearance of *Salmonella* in experimentally infected chickens and simultaneously resistance selection in *Salmonella* and coliforms were evaluated in three treatment groups (100, PK/PD designed dosage of 4, 0.1 mg/kg b.w.) and a control group. Treatment duration was three rounds of 7-day treatment alternated with 7-day withdrawal. Results showed that 100 mg/kg b.w. of enrofloxacin completely eradicated *Salmonella*, but resistant coliforms (4.0–60.8%) were selected from the end of the second round’s withdrawal period till the end of the experiment (days 28–42). PK/PD based dosage (4 mg/kg b.w.) effectively reduced *Salmonella* for the first treatment duration. However upon cessation of medication, *Salmonella* repopulated chickens and persisted till the end with reduced susceptibility (MIC_CIP_ = 0.03–0.25 mg/L). Low frequency (5–9.5%) of resistant coliforms was selected (days 39–42). Enrofloxacin at dosage of 0.1 mg/kg b.w. was not able to eliminate *Salmonella* and selected coliforms with slight decreased susceptibility (MIC_ENR_ = 0.25 mg/L). In conclusion, short time treatment (7 days) of enrofloxacin at high dosage (100 mg/kg b.w.) could be effective in treating *Salmonella* infection while minimizing resistance selection in both *Salmonella* and coliforms.

## Introduction

As a zoonotic foodborne pathogen, *Salmonella enterica* has been widely recognized as one of the most common causes of gastroenteritis in humans^1,2^. Fluoroquinolones (FQs) are important drugs for treating salmonellosis in both humans and animals^3–6^. However, development of antimicrobial resistance in bacteria may parallel the use of fluoroquinolones. Contribution of veterinary use of FQs to the resistance development in animal and human pathogens remains a major public concern^7–9^. Several studies indicated that FQ-resistant pathogens may arise de novo in animals from susceptible progenitors and be transmitted to humans via the food supply, causing potentially life-threatening infections^10–12^. Antimicrobial resistance in food-producing animals deserves special attention, especially clinically most critical antimicrobials (i.e. fluoroquinolones and cephalosporins).

In order to preserve the effectiveness of FQs, next to reducing the antimicrobial consumption, optimizing dosage regimens can be a suitable strategy to minimize antimicrobial resistance development without jeopardizing therapy efficacy and outcome. Pharmacokinetics/Pharmacodynamics (PK/PD) integration and animal infection models play essential roles in designing dosage schedule and bridge the gap between pharmacokinetics of antimicrobial drugs in target animal species and pharmacodynamics of their action on target pathogens^13^. The pharmacokinetics of FQs have been extensively studied in different animal species^14–18^, however the PK/PD model of enrofloxacin against *Salmonella* Typhimurium infection in chickens has not been established. Moreover, few studies validated the actual clinical efficacy and monitored the resistance selection in bacteria under the antibiotic pressure of PK/PD designed dosage. As the bacteria killing activity of enrofloxacin has been shown to be concentration-dependent, the impacts of enrofloxacin at highest non-toxic dosage of 100 mg/kg body weight (b.w.)^19^ and carry-over dosage of 0.1 mg/kg b.w. (due to contamination of feed with antibiotics, caused by carry-over of medicated feed to regular feed, or by left-over quantities in the drinking-water system) were also evaluated in this study.

The *in vivo* dynamics of the emergence of resistant bacteria and how bacteria respond to antibiotics under a mixture of stresses imposed by animal gastro-intestinal (GI) tract^20^ have not been clearly revealed. In addition, usage of antimicrobials selects for resistance not only in pathogenic bacteria but also in commensal bacteria which may make up a reservoir from where resistant clones can emerge and transfer resistance genes to any incoming pathogens or to themselves^21^, most published studies focused on a single bacterial species. In view of the above, the aim of this study was to compare the impacts of different dosage regimens of enrofloxacin against *Salmonella* Typhimurium infection in chickens and explore the best dosage regimen which could maintain or improve clinical efficacy but reduce selection of decreased susceptibility/resistance in both *Salmonella* Typhimurium and coliforms. For this purpose, a PK/PD model of enrofloxacin in chicken was established to optimize the dosage regimen. The effects of enrofloxacin dosages on clinical efficacy and resistance selection in *Salmonella* Typhimurium and coliforms were evaluated in three treatment groups (100 mg/kg b.w., PK/PD designed dosage of 4 mg/kg b.w., 0.1 mg/kg b.w.) and a non-treatment group. The treatment duration was three rounds of 7-day treatment alternated with 7-day withdrawal. The 7-day medication was set to investigate the clinical efficacy of enrofloxacin against *Salmonella* infection in chicken, as the manufacturer’s label directions for the use of enrofloxacin against salmonellosis in chickens recommends 5–10 days’ administration. The withdrawal period was set to 7 days for detecting the fitness (colonization) of *Salmonella* and coliforms in the absence of antibiotic selective pressure, as Landoni *et al*.^6^ found the withdrawal time of enrofloxacin to be 3–7 days. In clinical settings, antimicrobials may be repeatedly used because of re-infection of pathogens after withdrawal of medication, so three rounds of 7 days medication followed by 7 days withdrawal were implemented in this study.

## Results

### Dosage designed by PK/PD model

**Figure 1:**
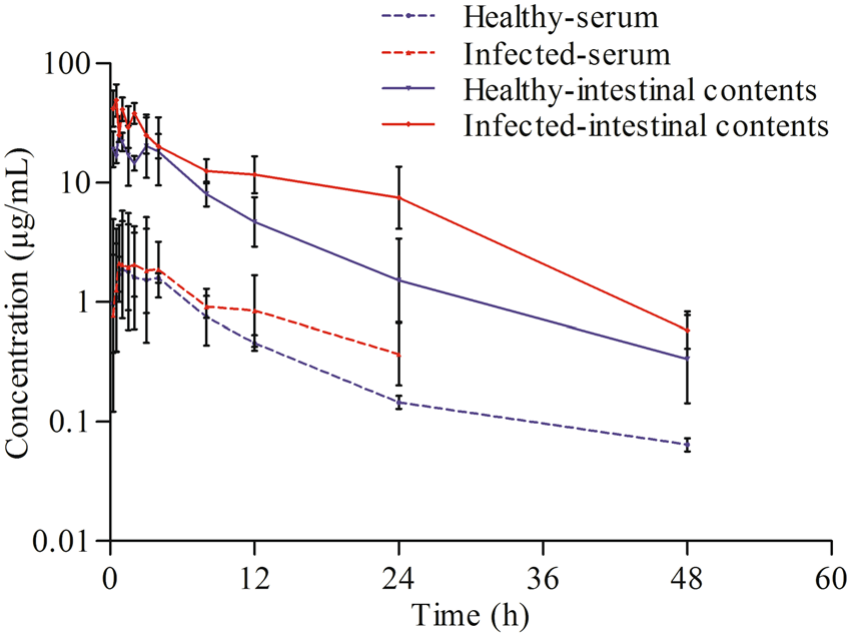
Semilogarithmic plots of the concentrations of enrofloxacin in serum and intestinal contents in healthy and infected chickens after oral administration at a dose of 10 mg/kg b.w. Values are means ± SDs (n = 5).

The mean ± standard deviation (SD) concentration-time profile of enrofloxacin in serum and intestinal contents of healthy and infected chickens are illustrated in Fig. 1. Pharmacokinetic parameters derived by non-compartmental analysis are presented in Table 1. Binding ratio of enrofloxacin to intestinal contents varied from 48.9 to 57.1% at concentration ranges of 0.2 to 200 mg/L. The mean binding ratio was 53% and the value of fu (fraction of drug not bound to feces) is 47%.

**Table 1:** Pharmacokinetic parameters of enrofloxacin in serum and intestinal contents of healthy and *Salmonella* Typhimurium infected chickens after oral administration of enrofloxacin at the dose of 10 mg/kg b.w.

Variable (units)	Serum		Intestinal contents	
	Healthy	infected	Healthy	infected
*C*_max_ (μg/mL)	1.873	2.108	28.17	48.99
*T*_max_ (h)	1.000	0.750	0.750	0.500
*T*_1/2_ (h)	9.052	8.848	9.632	8.566
AUC_0-last_ (μg h/mL)	19.31	23.46	210.7	448.9
AUC_0–24_ (μg h/mL)	16.80	23.46	188.6	351.8
AUMC_0-last_ (μg h/mL)	193.1	193.4	1854	5488
MRT _last_(h)	10.00	8.244	8.793	12.23
Cl_B_/*F* (L/kg)	0.496	0.355	0.046	0.022

Chickens were killed to obtain the serum and intestinal contents, so values are calculated from mean concentrations from five chickens at each time point. *C*_max_, maximum serum concentration; *T*_max_, time of maximum serum concentration; *T*_1/2_, elimination half-life; AUC_0-last_, area under the concentration-time curves from 0 h to last detection time point; AUC_0–24_, area under the concentration-time curves from 0 h to 24 h; AUMC_0–24_, area under first moment curve to 24 h; MRT _last_, mean residence time to last sampling time; Cl_B_/*F*, clearance scaled to bioavailability.

**Figure 2:**
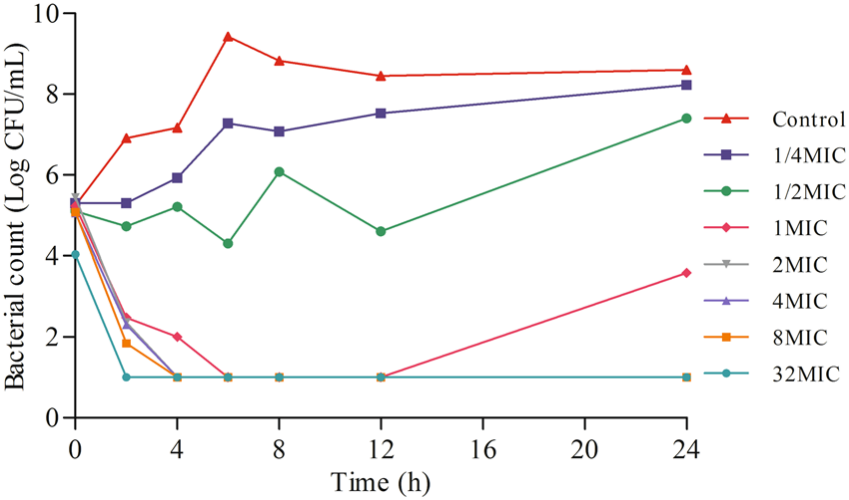
*In vitro* antibacterial activity of enrofloxacin against *Salmonella* Typhimurium CVCC541 in MH broth.

Minimal inhibitory concentration (MIC) distribution of 135 strains of *Salmonella* to enrofloxacin is illustrated in Supplementary Figure S1. MIC_50_ and MIC_90_ of enrofloxacin against these field *Salmonella* isolates were 0.12 mg/L and 16 mg/L, respectively. The MIC and minimal bactericidal concentration (MBC) values of enrofloxain against *Salmonella* Typhimurium CVCC541 were higher in intestinal contents (0.12 mg/L and 0.25 mg/L) than in MH broth (0.06 mg/L and 0.12 mg/L). The mutant prevention concentration (MPC) of *Salmonella* Typhimurium CVCC541 to enrofloxacin in MH broth was 0.8 mg/L. Post antibiotic effect was enhanced as the drug concentration and incubation time increased. *In vitro* time-kill curves, as illustrated in Fig. 2, demonstrated a concentration-dependent antibacterial activity of enrofloxacin against *Salmonella* Typhimurium CVCC541 in MH broth. *Ex vivo* antibacterial activities of enrofloxacin in intestinal contents of healthy and infected chickens are illustrated in Supplementary Figure S2. The results showed that for samples collected between 0.25 h and 24 h, enrofloxacin exerted prominent bactericidal effect. Whereas for the samples collected at 48 h, only slight inhibitory effects were observed initially and followed with a re-growth after 4 h incubation.

The PK/PD integration indices of C_max_/MIC, AUC_24h_/MIC, C_max_/MPC and AUC_24h_/MPC of enrofloxacin against *Salmonella* Typhimurium CVCC541 in intestinal contents are listed in Supplementary Table S1. Graphs depicting the relationship between bacterial counts and AUC_24h_/MIC values in intestinal contents of healthy and infected chickens are illustrated in Supplementary Figure S3. PK/PD modeled data and predicted dosages of enrofloxacin are presented in Table 2. The AUC_24h_/MIC values that required for bacteriostatic, bactericidal activity and bacterial eradication effects of intestinal contents in infected chickens were 538.64, 719.33 and 789.97 h, respectively. According to the equation (1), the optimized dosage of enrofloxacin against *Salmonella* Typhimurium CVCC541 infection in chicken for bactericidal activity was 4 mg/kg b.w.

**Table 2:** PK/PD modelling of *ex vivo* data of enrofloxacin in healthy and *Salmonella* infected chickens after administration of 10 mg/kg b.w. of enrofloxacin.

Parameters	Unit	Healthy		Infected	
		Value	Dosage(mg/kg)	Value	Dosage(mg/kg)
Log E_0_	CFU/mL	−6.68		−7.01	
Log E_max_	CFU/mL	1.46		2.14	
Slope (N)	—	9.40		4.96	
AUC_24_/MIC EC_50_	h	465.15		684.21	
AUC_24_/MIC for bacteriostatic action (E = 0)	h	395.67	2.22	538.64	3.03
AUC_24_/MIC for bactericidal action (E = −3)	h	474.76	2.67	719.33	4.04
AUC_24_/MIC for bacterial eradication (E = −4)	h	501.73	2.82	789.97	4.44

E_0_: difference in bacterial count in control sample (without enrofloxacin) between 0 and 24 h; E_max_: difference in bacterial count in sample incubated with enrofloxacin between 0 and 24 h, when the detection limit is reached; N: slope of the AUC_24h_/MIC-response curve; AUC_24_/MIC EC_50_: AUC_24_/MIC of drug producing 50% of the maximum antibacterial effect;

### Clinical efficacy of enrofloxacin against *Salmonella* Typhimurium at different doses

The clinical efficacy of different dosages of enrofloxacin against *Salmonella* Typhimurium was evaluated in three treatment groups (100 mg/kg b.w., PK/PD designed dosage of 4 mg/kg b.w., 0.1 mg/kg b.w.) and a non-treatment group.

A preliminary experiment was conducted to test the optimum inoculum of the challenge strain (*Salmonella* Typhimurium CVCC541). The results showed that 10^8^ CFU is the optimum inoculums, in which group the clinical signs of depression, diarrhoea, weakness were clearly observed after 12 h post infection, and no chicken died. In the group of 10^6^ CFU inoculums, the clinical signs of the chicken were not as evident as that in the group of 10^8^ CFU inoculums. In the group of 10^10^ CFU inoculums, three out of five chickens died within 24 h post infection.

For the clinical efficacy study, during the adaptation period, no chicken showed any clinical symptoms. All the chickens were negative for *Salmonella* prior to infection, as determined by culturing cloacal swabs. Inoculation of chicks with *Salmonella* Typhimurium CVCC541 induced severe clinical signs including depression, diarrhoea, weakness and moribund status within 48 h post infection. But none of the birds died throughout the course of the experiment. After initiation of the enrofloxacin treatment, severe clinical signs were absent from the chickens of 100 mg/kg b.w. and 4 mg/kg b.w. groups, while chickens of 0.1 mg/kg b.w. dosage group and non-medicated group recovered gradually as the chicken aged.

**Figure 3:**
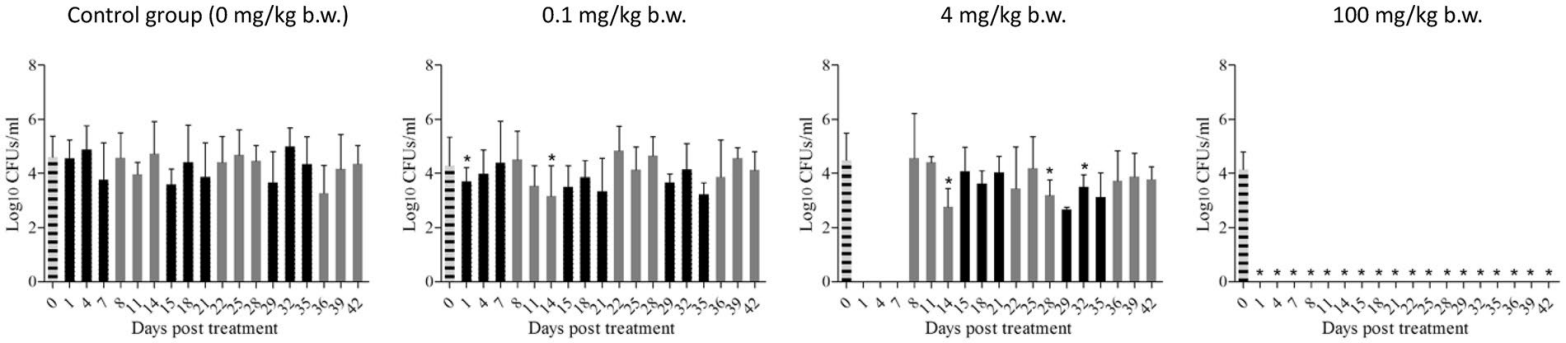
Effect of different dosages of enrofloxacin on the total viable counts of *Salmonella* shedding from chickens. Values are means ± SDs (n = 5). Black columns represent time points for treatment durations, grey columns represent time points for withdrawal periods, striped columns represent time points prior to medication (day 0). *mean values significantly different from those for the control group (*p* < 0.05).

Since *Salmonella* Typhimurium infection induced acute outbreaks exhibiting clinical disease mainly in chicks younger than 2 weeks old, infection was self-limiting and resulted in asymptomatic intestinal infections in elder chickens, so the clinical efficacy was also determined by enumerating variable counts of *Salmonella* Typhimurium in chicken feces. All the birds inoculated with *Salmonella* Typhimurium CVCC541 were quickly colonized and shed the organism at a level on average of 1.3 × 10^5^ CFU/g feces per bird before enrofloxacin treatment. Continuous shedding of *Salmonella* Typhimurium was observed in the control group throughout the sampling period at a relative stable level (10^5^ CFU/g feces) (Fig. 3). Enrofloxacin at dosage of 0.1 mg/kg b.w. did not have any impact on the overall number of *Salmonella* Typhimurium in chicken’s GI tract and the colonization level was not significantly different from the control group for most time points (except days 1 and 14 post medication). For the PK/PD designed dosage group, *Salmonella* Typhimurium was suppressed and the counts were reduced below the detection limit (100 CFU/g of feces) during the first 7-day treatment period. But upon cessation of the treatment, *Salmonella* Typhimurium reemerged, reverted back to the normal level and persisted till the end. Enrofloxacin dosing at a rate of 100 mg/kg b.w. virtually eradicated *Salmonella* Typhimurium from chicken’s GI tract immediately after administration and no *Salmonella* Typhimurium was recovered from chicken feces throughout the following experiment (Supplementary Table S2).

### Resistance selection in *Salmonella* Typhimurium

Despite different doses of enrofloxacin treatment, no resistant *Salmonella* Typhimurium (i.e., ability to grow on CHROMagar *Salmonella* plates with 1 mg/L ciprofloxacin) were detected in any of the treated chickens during the entire experiment by both differential plating and MIC testing. But in the PK/PD dosage group, reduced susceptibility of *Salmonella* Typhimurium was observed based on the elevated MICs of *Salmonella* Typhimurium to ciprofloxacin, increasing from 0.015 mg/L (before treatment) to 0.03–0.25 mg/L (post treatment). None of the birds in the control and low dosage group gave rise to *Salmonella* Typhimurium with reduced susceptibility.

### Resistance selection in coliforms

#### Control group

**Figure 4:**
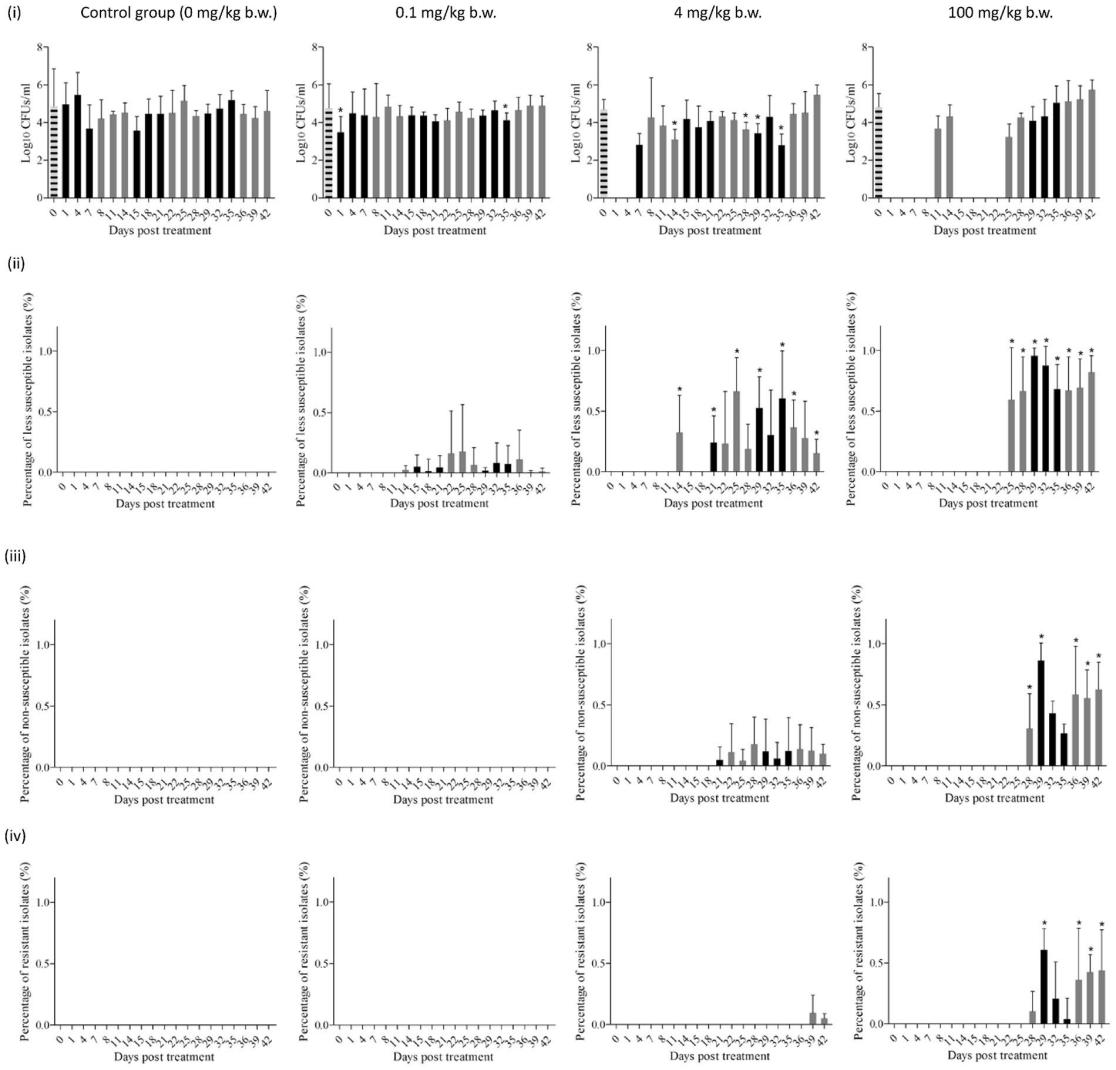
Effect of different dosages of enrofloxacin on resistance development in chicken fecal coliforms. (**i**) total counts of coliforms during experiment; (**ii**) levels of less susceptible (grown on MacConkey plates containing 0.125 mg/L enrofloxacin) coliforms during experiment; (**iii**) levels of non-susceptible (grown on MacConkey plates containing 0.25 mg/L enrofloxacin) coliforms during experiment; (**iv**) levels of resistant (grown on MacConkey plates containing 2 mg/L enrofloxacin) coliforms during experiment. Values are means ± SDs (n = 5). Black columns represent time points for treatment durations, grey columns represent time points for withdrawal periods, striped columns represent time points prior to medication (day 0). *mean values significantly different from those for the control group (*p* < 0.05).

The feces collected from the chickens before start of the treatment contained on average of 3 × 10^6^ CFU/g coliforms per chicken and such colonization levels were maintained during the entire experiment. MICs of 200 isolates of coliforms collected prior to medication were consistently 0.06–0.12 mg/L, representing no enrofloxacin resistance background in the commensal flora of chick’s intestine. Moreover, titration of the cloacal swabs on enrofloxacin-supplemented agar plates (i.e., 0.125 mg/L of agar, 0.25 mg/L of agar and 2 mg/L of agar) did not show any growth of less susceptible or resistant isolates at any time during the experiment (Fig. 4).

#### Low dosage group

For the low dosage group, no changes of the total coliform counts were detected during the course of the studies. Coliforms with decreased susceptibility were selected from the MacConkey plates containing 0.125 mg/L enrofloxacin from day 14 onwards at frequencies of 0.6–17% (Supplementary Table S3). The MIC_ENR_ values of these coliform isolates varied from 0.06 to 0.25 mg/L. No colonies were detected on MacConkey plates containing 0.25 or 2 mg/L enrofloxacin.

#### PK/PD dosage group

Within the first 7-day treatment of enrofloxacin at dosage of 4 mg/kg, fecal coliforms were eliminated or suppressed below the detection limit of 100 CFU/g feces. But coliforms counts recovered on the 7th day of the first treatment period, gradually increased to the initial level of 10^6^ CFU/g feces and persisted throughout the rest of the experimental period (Fig. 4). The frequencies of less susceptible isolates (recovered from MacConkey plates containing 0.125 mg/L enrofloxacin) were 32.2% on day 14 and 15.1–66.1% between days 21–42. Non-susceptible coliforms, which could grow on MacConkey plates containing 0.25 mg/L enrofloxacin, were selected from day 21 onwards with percentages ranging from 4.2 to 17.8% (Supplementary Table S3). At the end of last withdrawal period (days 39–42), low frequencies (5–9.5%) of resistant coliforms (ability to grow on MacConkey plates containing 2 mg/L enrofloxacin) were selected.

#### High dosage group

When dosing at rate of 100 mg/kg b.w., enrofloxacin induced a rapid reduction of the coliforms counts below the detection limit for the first 7-day treatment period. But on days 4–7 of the following withdrawal period, coliforms recovered back to the pre-treatment level steadily (Fig. 4). The second 7-day treatment of enrofloxacin cleared coliforms again. On the 4th day of the second withdrawal period, coliforms gradually recovered and persisted till the end. High percentages (66–95%) of coliforms with reduced susceptibility appeared on days 32–42. Non-susceptible (26.5–86.2%) and resistant (4–60.8%) coliforms were frequently selected from day 28 onwards (Supplementary Table S3).

#### MIC and target mutations of *Escherichia coli*

Of all the 1034 strains of coliforms, randomly picked from different MacConkey plates, 330 isolates were identified to be *Escherichia coli* (*E. coli*). They were all selected on MacConkey plates containing 2 mg/L enrofloxacin. And all the *E. coli* were isolated from chickens of PK/PD based dosage group and high dosage group. MIC_ENR_ values of the *E. coli* were ranged from 8–256 mg/L which were above the resistance breakpoint of 2 mg/L. So all the 330 strains of *E. coli* were resistant to enrofloxacin. Double mutations on GyrA (S83L, D87N) and a single mutation on ParC (S80I) were observed for all the resistant *E. coli*. No mutations were found on GyrB. A single mutation (S458A) was observed on ParE in 302 of 330 strains of *E. coli*, which showed high level resistance to enrofloxacin (MIC_ENR_ ⩾ 128 mg/L).

## Discussion

In this study, by applying the method of PK/PD integration and modeling, we optimized the dosage regimen of enrofloxain against susceptible *Salmonella* Typhimurium infection in chickens: enrofloxacin at dosage of 4 mg/kg b.w. for 7 days. The clinical efficacy on chicken and resistance selection in *Salmonella* Typhimurium and commensal coliforms of enrofloxacin were subsequently evaluated in three treatment groups (100 mg/kg b.w., PK/PD designed dosage of 4 mg/kg b.w., 0.1 mg/kg b.w.) and a non-treatment group. High dosage of enrofloxacin virtually eradicated artificially infected *Salmonella* Typhimurium immediately, but selected resistant coliforms from the end of the second round’s withdrawal period till the end of the experiment (days 28–42). PK/PD based dosage was effective against *Salmonella* Typhimurium for the first 7-day treatment period. However upon cessation of medication, *Salmonella* Typhimurium repopulated in chickens and persisted till the end with reduced susceptibility to ciprofloxacin. Low frequency of resistant coliforms was selected at the end of experiment. Enrofloxacin at the dosage of 0.1 mg/kg b.w. was not able to eliminate *Salmonella* Typhimurium and selected coliforms with slightly decreased susceptibility to enrofloxacin.

According to the manufacturer label directions of enrofloxacin (Baytril 10% oral solution), for treatment of Salmonellosis in chicken, enrofloxacin is recommended to be administered at 10 mg/kg b.w. (50 ppm) for 5–10 days. Several researchers have investigated the clinical efficacy of enrofloxacin against *Salmonella* and resistance selection in *Salmonella* and *E. coli* at the clinically recommended dosage. Despite different treatment protocols used, main findings in this study were consistent with the results reported by other researchers. Barrow *et al*.^22^ reported that enrofloxacin treatment at a clinical recommended dosage (50ppm) for 11 days largely cured an experimental infection with *S. Enteritidis* in chickens, but it caused the commensal *E. coli* population resistant to fluoroquinolone. In a study by Wiuff *et al*.^23^, enrofloxacin treatment of pigs induced fluoroquinolone resistance in the resident coliforms and selected for an artificially introduced strain of *Salmonella* Typhimurium with decreased susceptibility to ciprofloxacin. A similar study^24^ reported that after administration with 1/8, 1/4, 1/2 and 1/1 of recommended dosage of enrofloxacin to *Salmonella* Typhimurium infected chickens, resistant *Salmonella* Typhimurium was selected, but the level was not as high as commensal *E. coli*.

It seems that the fluoroquinolone resistance in *Salmonella* Typhimurium is not as frequent as that in *Escherichia coli*. Based on the latest European Union summary report on antimicrobial resistance in zoonotic and indicator bacteria from humans, animals and food in 2014^25^, regarding indicator commensal *Escherichia coli* in broilers, the highest overall ‘microbiological’ resistance levels observed at the reporting MS group level were to ciprofloxacin (65.7%). *Salmonella* isolates from broiler flocks exhibited high-level resistance to ciprofloxacin, especially in serovars Enteritidis (24.6%) and Infantis (92.7%). However, different serovars of *Salmonella* behave differently in relation to resistance development. FQ-resistance in *Salmonella* Typhimurium from broilers was not fully introduced in the report. Among all serovars, isolates resistant to ciprofloxacin, but not to nalidixic acid, were observed, probably indicating an increasing occurrence of plasmid-mediated quinolone resistance^25^. In the present study, chickens were raised in separate close-down isolators, the possibility of acquired FQ-mechanism of plasmid-mediated quinolone resistance gene was minimized. We did not select *Salmonella* Typhimurium mutants *in vivo* that reached the level of resistance to ciprofloxacin. This is obviously not due to limited bioavailability of fluoroquinolone in chicken’s gut (e.g., through binding to fecal material), since fully resistant *E. coli* could be selected under the same *in vivo* conditions. Two possible explanations are suggested to account for this phenomenon. Firstly, the higher numbers of *E.coli* in the chick’s intestine, indicated by the higher fecal estimates, probably facilitates the development of enrofloxacin resistance in the *E. coli* populations. Secondly, resistance mechanisms mediated by target alterations are believed to induce a fitness cost in *Salmonella* ^24,26^. The mechanisms leading to a high level of FQ-resistance in *Salmonella* could be deleterious and result in *Salmonella* to be counterselected under *in vivo* conditions^24^. Nucleoid partitioning defects have already been reported in *S*. Typhimurium strains mutated genes that code for topoisomerase IV^24^.

For the parameters derived by pharmacokinetics study, the C_max_ of intestinal enrofloxacin concentrations were 28.17 μg/g and 48.99 μg/g for healthy group and infected group respectively, which are comparable to other reports^27–29^. In general, enrofloxacin concentrations in intestinal contents were much higher as compared to plasma concentrations. Unlike in other animal species, the biotransformation of enrofloxacin to ciprofloxacin was limited (7%) in poultry and added only minimally to the antimicrobial effect of enrofloxacin, so the effect of ciprofloxacin was not considered in this study. Bacterial infections have been shown to alter the pharmacokinetics of drugs, including fluoroquinolones^30,31^. In the present study, the C_max_, AUC and MRT of enrofloxacin were higher in the infected chickens than in the healthy group. And enrofloxacin absorption following oral administration is largely incomplete in chickens, as a consequence, these high concentrations of unabsorbed drugs in the intestine are likely to exert a great pressure on the gut microflora and lead to the eradication of the pathogenic *Salmonella* Typhimurium from the gut.

**Figure 5:**
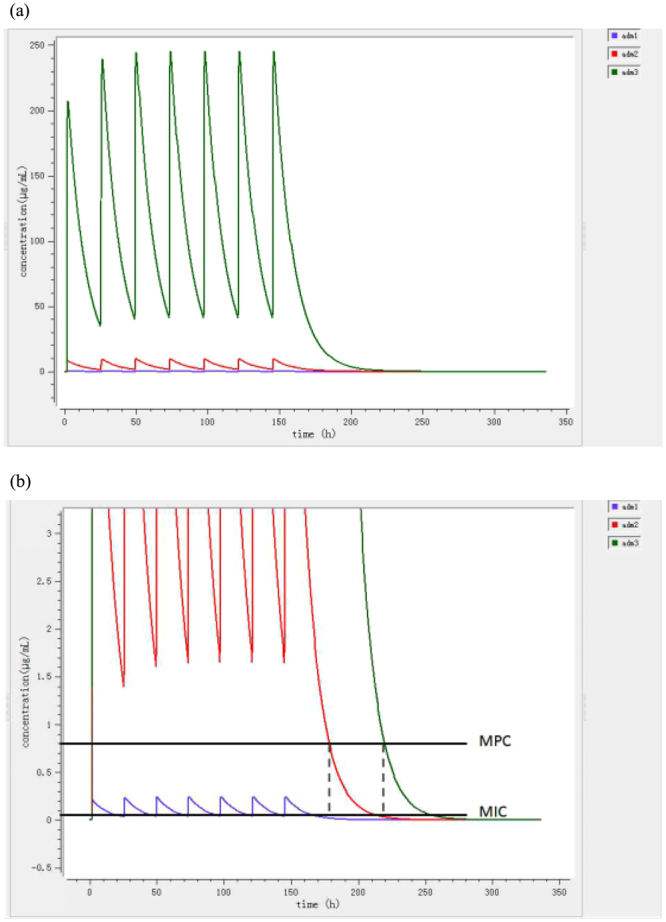
Prediction of the enrofloxacin concentrations in intestinal contents of chickens treated with three dosage of enrofloxacin (blue: 0.1 mg/kg b.w.; red: 4 mg/kg b.w.; green: 100 mg/kg b.w.) by Mlxplore software. The upper window (**a**) shows the whole picture, the lower window (**b**) shows the enlarged version.

The breakpoints of C_max_/MIC ⩾ 10 and AUC/MIC ⩾ 125 are indicators for therapeutic efficacy of fluoroquinolones and for the minimization of resistance selection in bacteria^29^. Based on prediction by the Mlxplore software (version 1.1.0, Lixoft, Orsay, France) (Fig. 5), the intestinal enrofloxacin concentrations of the high dosage group would reach about 40~250 mg/L for the 7-day treatment period, which is above the MPC (0.8 mg/L) and high enough to eradicate all the *Salmonella* Typhimurium from chickens GI tract. In the PK/PD based dosage group, the C_max_ of enrofloxacin would be about 10 mg/L for the first 7-day treatment period, which could effectively reduce *Salmonella* Typhimurium blow the detection limit. But in the withdrawal period of time, the concentration decreased and fell into the mutant selection window (MSW). *Salmonella* Typhimurium with reduced susceptibility may be selected and survive in the chicken intestine or in the isolators at undetectable amount, then repopulate in the chickens. For the low dosage group, the enrofloxacin C_max_ is about 0.2 mg/L and the mean binding ratio of enrofloxacin to feces is 53%, so the unbound fraction of enrofloxacin is about 0.1 mg/L, which is lower than the MIC of *Salmonella* Typhimurium CVCC541 in intestinal contents (0.12 mg/L). It is also reported that the low tension of oxygen in the intestine might reduce the *in vivo* activity of fluoroquinolones^23^. Thus, enrofloxacin at dosage of 0.1 mg/kg b.w. did not have any inhibition impact on the *Salmonella* Typhimurium. The PK/PD based dosage is effective for the clinical cure, but from the point of microbiological cure, it does not achieve an ideal treatment outcome since reinfection of *Samonella* Typhimurium with decreased susceptibility occurs. PK/PD dosages which target at higher antibacterial effect (virtual eradication) or based on the PD parameter of MPC might be effective in clinical efficacy and minimizing resistance selection. The effectiveness of these kinds of dosages needs to be verified in further studies.

The resistance level and frequency of coliforms were positively correlated with the administration dosage of enrofloxacin. In the low dosage group, the frequency of less susceptible isolates was 0.6–17%, while it was 15–66% for PK/PD dosage group and 66–95% for high dosage group. For the resistant coliforms, the frequency was 5–9.5% for PKPD dosage group and 36–43% for high dosage group. This was presumably due to the background bacterial counts in the fecal samples were drastically reduced by the high dose of enrofloxacin treatment. The antibiotic treatment diminished not only the FQ-sensitive *Salmonella* Typhimurium but also other competing bacterial flora in the gut, resulting in a favorable environment for the rapid propagation and transmission of FQ-resistant coliforms in the experimental chickens.

Another important finding of this study is that the FQ-resistance of coliforms develops in a stepwise manner. In PK/PD dosage group, the less susceptible isolates were observed on day 14, non-susceptible isolates emerged on day 21 and resistant coliforms were selected from day 39 onwards. In high dosage group, the less susceptible isolates were obtained on day 25, non-susceptible and resistant coliforms were isolated from day 28 onwards. These findings were consistent with other reports^32–34^. Drlica *et al*.^35^ found that *de novo* quinolone resistance often develops in a gradual, stepwise manner, usually from the accumulation of mutations in the *gyr* and *par* genes that individually lower the susceptibility by modest increments and requires prolonged exposure to the selection pressure. A first step mutation usually leads to reduced susceptibility, after which successive mutations possibly result in resistance. And also in this study, most of the resistant coliforms were selected during the later course of antibiotic treatment and antibiotic cessation periods. Similar observations have been made previously^36^.

As an indicator organism, *E. coli* is a good candidate for studies of antibiotic resistance mechanisms, so it was chosen as the target organism for the further phenotypic and genotypic resistance study. Our data demonstrated that the presence of two mutations in GyrA (S83L, D87N) and one mutation in ParC (S80I) was associated with resistance to enrofloxacin (MIC ⩾ 8 mg/L) in the *E. coli*, and an additional mutation (S458A) on ParE was involved in higher level resistance (MIC_ENR_ ⩾ 128 mg/L), which was consistent with previously published data^37,38^. Mutations in DNA gyrase and topoisomerase IV are the most prevalent and important mechanisms mediating the fluoroquinolone resistance in *E. coli*. It is reported that at least three mutations, two of which must be in *gyrA*, are required to achieve CLSI-classified clinical resistance and the MICs for quadruple mutants are_10-fold higher than those for triple mutants^39^. Moreover, since the resistant *E. coli* exhibited a wide range of resistance levels (MIC = 8–256 mg/L), indicating the presence of additional resistance mechanisms (including decreased permeability to quinolones through modifications of the outer membrane proteins or an active efflux mechanism or carry on of plasmid mediated quinolone resistance genes) which needs to be addressed by further investigations.

When assessing the effect of different dosages of enrofloxacin on clinical efficacy and resistance selection, both dosage and duration of therapy play a part. Since enrofloxacin exerts a concentration-dependent antibacterial activity, it is hypothesized that higher plasma and intestinal concentrations might succeed in a better bacterial killing. Concentrations of enrofloxacin need to be adequate to either eradicate the existing bacterial population in GI tract or (at least) to reduce its size to the point where the host’s defense mechanisms can successfully control and eliminate the remaining pathogens. Our results showed that high dosage (100 mg/kg b.w.) of enrofloxacin provided a sufficient concentration of active drug in the intestinal contents to eradicate the *Salmonella* Typhimurium. Moreover it supports the view of “hit hard” expounded by Ehrlich and “the highest tolerated antibiotic dose” principle^40,41^. Findings from this study indicate that long term antibiotic treatment seems to be the major trigger for the development of resistance in coliforms, since resistant coliforms are selected from the end of the second round’s withdrawal period (day 28) in high dosage group and the end of the third round’s withdrawal period (day 39) in PK/PD dosage group. Additionally, several studies suggested that shorter treatment duration selected for lower percentages of strains with reduced susceptibility^42–44^. So enrofloxacin treatment at a high dose for short course may be effective for clinical therapy, meanwhile selecting for less resistance in both *Salmonella* Typhimurium and coliforms. The authors would like to stress out that administration of a tenfold increased dose without reconsidering the duration of the therapy, might lead to improper usage of fluoroquinolones. And also, the problems of drug residues and withdrawal time should be considered in such cases. In the present study, no adverse effects were observed on chickens administered at such high dose of enrofloxacin, and this was consistent with the toxicity data for this drug. More extensive studies of the possible toxic effects on growing chickens, drug residues and withdrawal time needs to be performed before the elevated dose is recommended for the clinical therapy.

In conclusion, short time treatment of enrofloxacin at a high dosage could be effective in treating *Salmonella* Typhimurium infection while selecting for less resistance in both *Salmonella* Typhimurium and coliforms in chickens. PK/PD designed dosage which targeted at higher antibacterial effect (virtual eradication) or based on MPC may achieve ideal treatment outcome when combining with hygiene procedure to reduce the occurrence of reinfection in treated chickens. This study firstly evaluates the effects of long-term treatment of different dosages of enrofloxacin on selection of resistance in both *Salmonella* Typhimurium and coliforms *in vivo* and offers insights into the rational use of FQs in animal husbandry.

## Materials and Methods

### Bacteria, antimicrobial and chemicals

The challenge isolate was *Salmonella* Typhimurium CVCC541, which was purchased from Chinese Veterinary Culture Collection. Cultures were grown overnight at 37 °C in Luria-Bertani (LB) broth prior to infection of chicks and for *in vitro* studies. Enrofloxacin (Baytril 10% oral solution) for dosing chickens was provided by Bayer HealthCare AG (Germany). Other antibiotics and organic solvents used were obtained from Dr. Ehrenstorfer (Augsburg, Germany).

### Design of dosage by PK/PD modeling

All animal experiments in this study were approved by the Animal Care Center, Hubei Science and Technology Agency in China (SYXK 2013–0044) and conducted according to the guidelines of the committee on the use and care of the laboratory animals in Hubei province.

One hundred and thirty healthy Cobb broilers at the age of thirty-day-old weighing 1.0–1.5 kg were equally allocated into two groups (group A and group B) and reared in separate rooms with *ad libitum* access to drinking water and antibiotic-free feed. After acclimatization for 7 days, each chicken in group A was inoculated with 10^8^ CFU *Salmonella* Typhimurium CVCC541 by gavage intubation. Group B was maintained as healthy control. Twenty-four hours after infection, each chicken of the two groups was administered an oral bolus of enrofloxacin (Baytril 10% oral solution) at the clinically recommended dosage of 10 mg/kg b.w. Five chickens from each group were euthanized to collect blood and intestinal content samples before medication and at time points of 0.25, 0.5, 0.75, 1, 1.5, 2, 3, 4, 8, 12, 24 and 48 h post medication. All the samples were assayed for enrofloxacin and ciprofloxacin by the high pressure liquid chromatography (HPLC) method with fluorescence detection essentially as described previously^45,46^. Enrofloxacin concentration-time data in serum and intestinal contents were analysed using the WinNonlin regression programme (version 5.2.1, Pharsight Corporation, Mountain View, CA, USA). Data for serum and intestinal contents were submitted to non-compartmental analysis using the statistical moment approach described by Perrier and Mayershhn^47^. The liner trapezoidal rule was used to calculate AUC values and area under the first moment curve (AUMC). The mean residence time (MRT) was determined as AUMC/AUC. Binding ratio of enrofloxacin to intestinal contents was determined by ultra-filtration method^48^.

Minimal inhibitory concentrations (MICs) of enrofloxacin against 135 strains of *Salmonella* were determined by agar dilution method according to guidelines of the Clinical and Laboratory Standards Institute^49^. All these 135 strains of *Salmonella* were isolated from cloacal swabs collected from different poultry farms in Henan, Hubei, and Hunan provinces of China between March 2010 and July 2011 and were stored in −80 °C in our lab. The detailed information of sample collection and *Salmonella* isolation were fully described in a previous published paper^50^. The MIC and minimal bactericidal concentration (MBC) of enrofloxacin against *Salmonella* Typhimurium CVCC541 in Mueller Hinton Broth (Hopebiol, Qingdao, China) and intestinal contents (obtained from healthy non-medicated chickens) were measured by microdilution method based on CLSI guidelines^49^. The mutant prevention concentration (MPC) and post antibiotic effect (PAE) of enrofloxacin against *Salmonella* Typhimurium CVCC541 were determined as previously described^51,52^. *In vitro* time-kill curve (in MH Broth) and *ex vivo* antibacterial activities (in intestinal contents obtained from chickens of group A and group B at different time points) of enrofloxacin against *Salmonella* Typhimurium CVCC541 were detected following the method described by Balaje^45^.

The surrogate markers of antimicrobial activity, C_max_/MIC, AUC_24h_/MIC, C_max_/MPC and AUC_24h_/MPC were calculated for intestinal contents of healthy and infected chickens, respectively. AUC_24h_/MIC data from *ex vivo* bacterial growth inhibition curves were modeled to the sigmoidal E_max_ equation. Three levels of antibacterial effect of enrofloxacin were quantified by determining AUC_24h_/MIC required for bacteriostatic, bactericidal and eradication action. Daily dosages were calculated according to the equation (1).

1 $$Dose=\frac{{(AU{C}_{24h}/MIC)}_{exvivo}\times MI{C}_{50}\times Clearance}{F\times fu}$$

where AUC_24h_/MIC = targeted end point for optimal efficacy, MIC = MIC for *Salmonella* Typhimurium CVCC541, MIC_50_ = concentration for inhibiting 50% of the field *Salmonella* isolates. F = bioavailability which is a measurement of the rate and extent to which a drug reaches at the site of action, *fu* = fraction of drug not bound to feces determined by ultra-filtration method.

### Clinical efficacy of enrofloxacin against *Salmonella* Typhimurium at different doses

The clinical efficacy of enrofloxacin against *Salmonella* Typhimurium was evaluated at dosages of 100, PK/PD designed dosage of 4, 0.1 mg/kg b.w. and a non-medicated group.

A preliminary experiment was conducted to test the optimum inoculum of the challenge strain (*Salmonella* Typhimurium CVCC541). Three inoculums of *Salmonella* (10^6^, 10^8^ and 10^10^ CFU) were challenged to three groups of chickens at their 4 days old. Each group consists of five chickens. For the formal clinical efficacy study, twenty specific-pathogen-free (SPF) chicks (1-day-old) were randomly separated into four groups of five each and reared in individual isolators. The number of chickens was calculated by resource equation method^53^. The value of E (Total number of animals − Total number of groups) was 16, which was between 10 and 20 and could be considered as appropriate.

All the chickens received non-medicated feed and water *ad libitum* and were monitored for conditions twice daily throughout the experiment. At 4 days old, all chicks were infected with *Salmonella* Typhimurium CVCC541 at the optimum inoculum by gastric gavage individually. At 8 days old, enrofloxacin (Baytril 10% oral solution) at the doses of 100 mg/kg b.w., 4 mg/kg b.w. (PK/PD based), 0.1 mg/kg b.w. and 0 mg/kg b.w. were administered to each group of chickens respectively by oral gavage for three 7-day treatments alternated with 7-day withdrawal periods. Cloacal swabs were taken on the day prior to medication (day 0) and at 1, 4, 7, 8, 11,14, 15, 18, 21, 22, 25, 28, 29, 32, 35, 36, 39 and 42 d post-start of the treatment. Each swab was weighed and emulsified in 1 mL of sterile saline, decimal dilutions were then steaked onto CHROMagar *Salmonella* agar plates (CHROMagar, France). *Salmonella* appearing typical purple colonies were counted after incubation at 37 °C for 24 h. Two colonies were randomly picked and stored in −80 °C for further identification and MIC test.

### Resistance selection in *Salmonella* Typhimurium and coliforms

Cloacal swabs collected in the clinical efficacy trial were also examined for the total coliform counts (TCC) and presence of fluoroquinolone-resistant *Salmonella* Typhimurium and coliforms. Aliquotes of serial 10-fold dilutions were spread onto MacConkey agar plates (Hopebiol, Qingdao, China) containing 0 mg/L, 0.125 mg/L, 0.25 mg/L and 2 mg/L of enrofloxacin for determination of TCC and coliforms with different levels of reduced antimicrobial susceptibility. This was repeated for resistant *Salmonella* Typhimurium on CHROMagar *Salmonella* agar plates with 1 mg/L ciprofloxacin. All the plates were incubated at 37 °C for 24 h and total colony counts were recorded. Two colonies, randomly selected from each plate, were further identified by biochemical tests and 16 S rRNA sequencing using primers (F: CCAGACTCCTACGGGAGGCAG, R: CGTATTACCGCGGCTGCTG). Antimicrobial susceptibilities of enrofloxacin against all the coliforms and *Salmonella* Typhimurium were determined by agar dilution method^49^. MICs of enrofloxacin were also determined for 200 strains of fecal coliforms isolated from clocal swabs of these 20 SPF chicks (ten isolates per chicken) at their 5-day-old (before medication) to check the resistance background of commensal flora. *E. coli* ATCC25922 was used as the quality-control organism. For all the resistant *E. coli* and *Salmonella* Typhimurium isolates, the *gyrA*, *gyrB*, *parC* and *parE* genes in the quinolone resistance determining region (QRDR) were amplified using the primers from Everett *et al*.^54^ and the amplification products were subjected to sequencing.

### Statistical analysis

Statistical analyses were undertaken using GraphPad InStat (GraphPad Software Inc., San Diego, CA). For the pharmacokinetic study, differences of the concentration of enrofloxacin in serum and intestinal contents between healthy and infected chickens were detected by unpaired *t* test. For the clinical efficacy experiment, differences of the treatment outcome (reflected by the viable counts of *Salmonella* Typhimurium) between each treatment group and the control group were examined by a one-way analysis of variance (ANOVA) and Dunnett test. For the resistance selection experiment, differences of variables (total counts of coliforms, percentage of less susceptible isolates, percentage of non-susceptible isolates and percentage of resistant isolates) between each treatment group and the control group were examined by a one-way analysis of variance (ANOVA) and Dunnett test. Differences were accepted as significant for *p* values <0.05.

### Data Availability

All data generated or analysed during this study are included in this published article (and its Supplementary Information files).

## Electronic supplementary material

Supplementary materials
