# Supplementary material for: The effects of different enrofloxacin dosages on clinical efficacy and resistance development in chickens experimentally infected with Salmonella Typhimurium
Source: Sci Rep. 2017 Sep 15;7:11676. doi: 10.1038/s41598-017-12294-7 (PMC5601478; doi:10.1038/s41598-017-12294-7)
Supplement: Supplementary file 1 — Supplementary materials [file 41598_2017_12294_MOESM1_ESM.pdf]

The effects of different enrofloxacin dosages on clinical efficacy and resistance development in chickens experimentally infected with *Salmonella* Typhimurium

Jun Li[1], Haihong Hao[2]*, Guyue Cheng[2], Xu Wang[2], Saeed Ahmed[1], Muhammad Abu Bakr Shabbir[1], Zhenli Liu[3], Menghong Dai[2]* & Zonghui Yuan[1, 2, 3]*

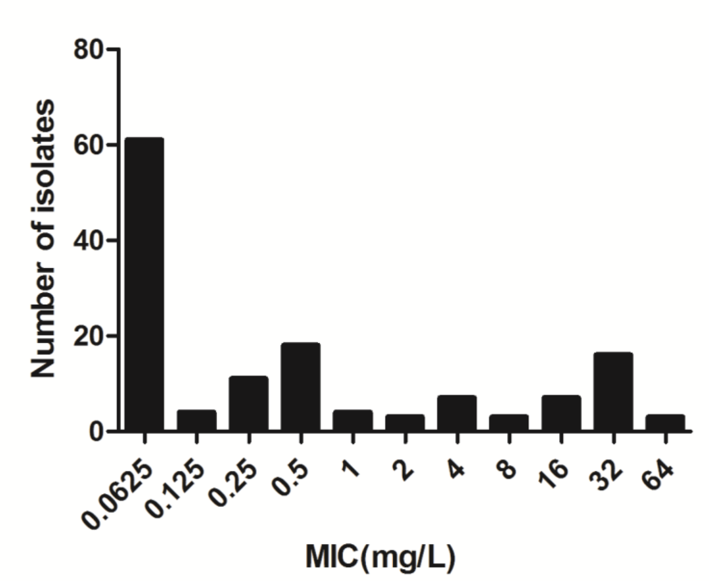

Supplementary Figure S1. MIC distribution of enrofloxacin against 135 strains of *Salmonella* isolates in MH broth.

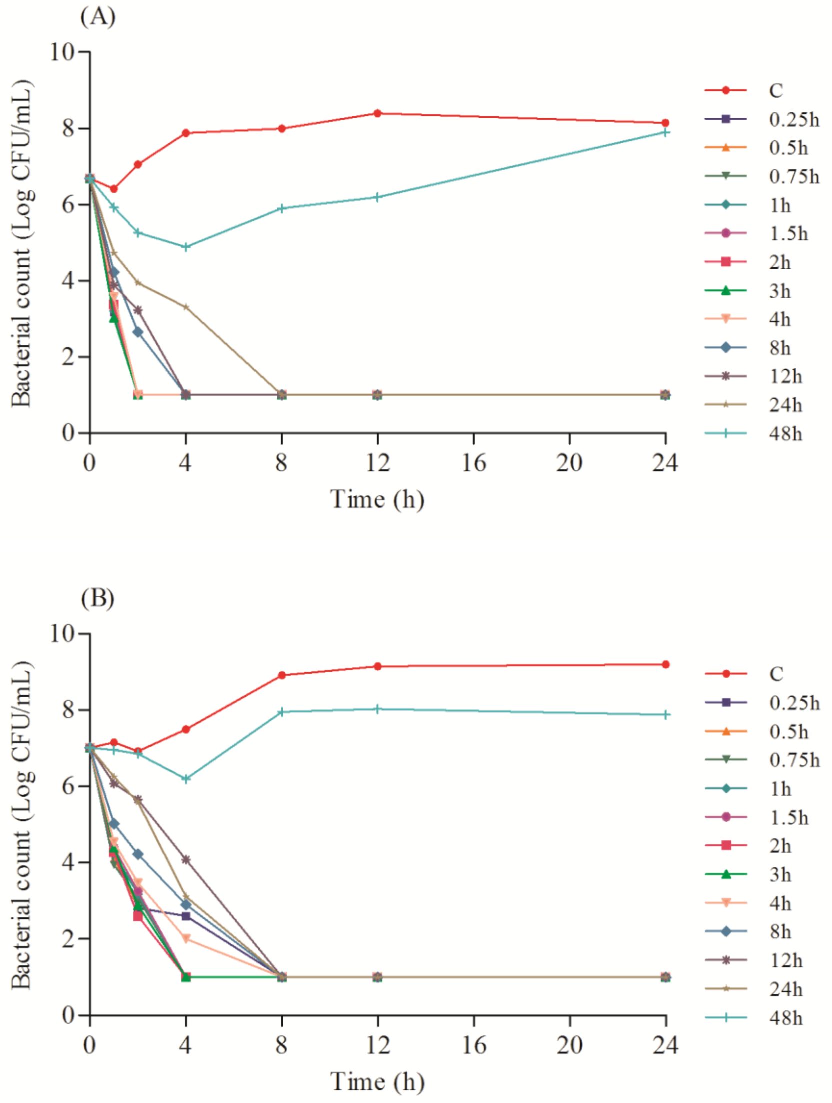

Supplementary Figure S2. *Ex vivo* antibacterial activities of enrofloxacin against *Salmonella* Typhimurium CVCC541in intestinal contents of healthy (A) and infected (B) chickens after oral administration at a dose of 10 mg/kg.

a

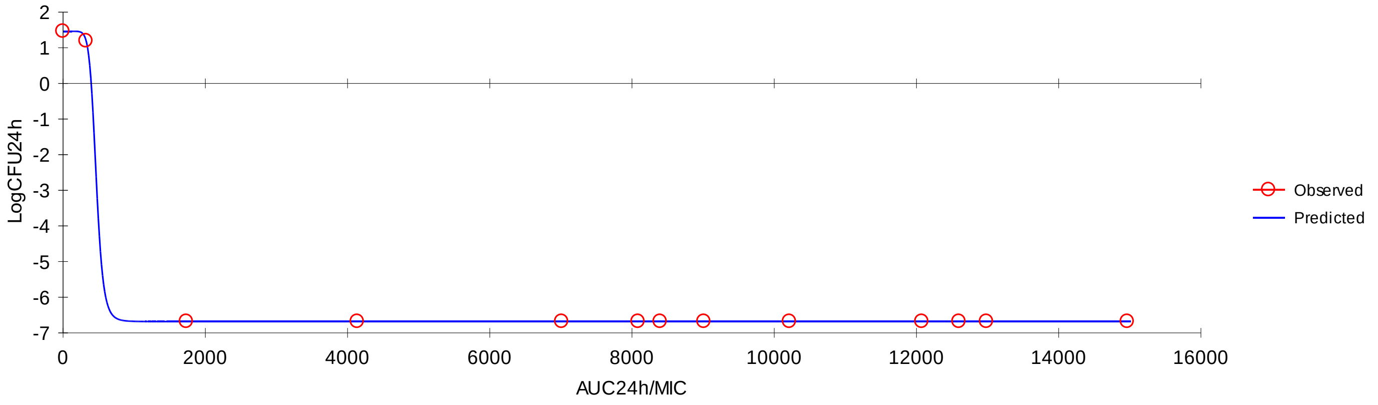

b

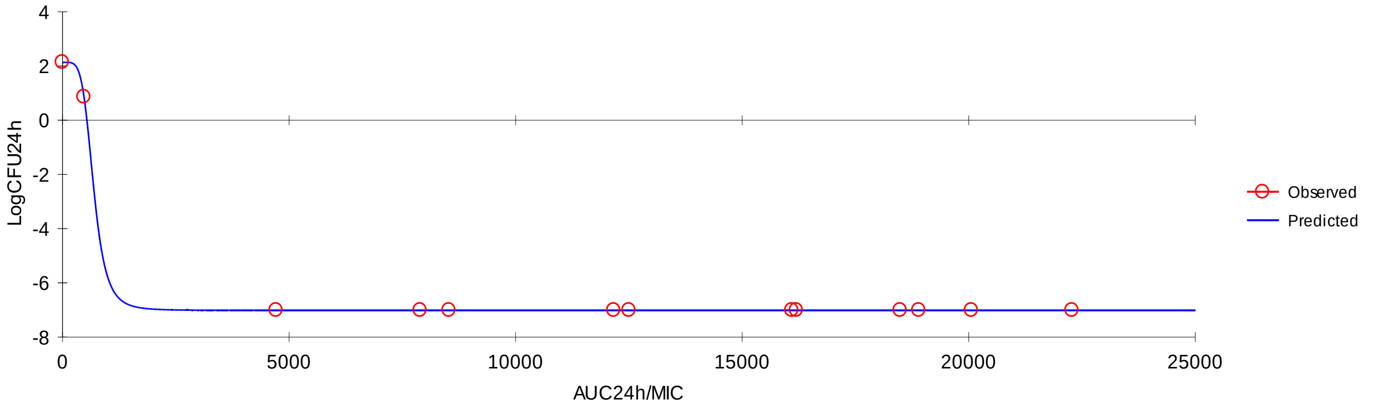

Supplementary Figure S3. Plots of *ex vivo* AUC_{24h}/MIC versus bacterial count (log_{10} CFU per milliliter) for *Salmonella* Typhimurium CVCC541 in intestinal contents of healthy (a) and infected (b) chickens. The curves are the best fit based on the sigmoid E_{max} equation. Each point represents mean value of five chickens.

Parameter	Unit	Healthy	Infected
AUC_{24h}/MIC	h	1572	2932
AUC_{24h}/MPC	h	235.8	439.8
C_{max}/MIC	-	234.8	408.3
C_{max}/MPC	-	35.21	61.24

Supplementary Table S1. PK/PD integration surrogate markers of enrofloxacin in intestinal contents of healthy and *Salmonella* infected chickens. AUC_{24h}/MIC, the area under the concentration-time curve over 24 h in steady state divided by the MIC; AUC_{24h}/MPC, the area under the concentration-time curve over 24 h in steady state divided by the MPC; C_{max}/MIC, maximum serum concentration divided by the MIC; C_{max}/MPC, maximum serum concentration divided by the MPC.

Dose (mg/kg)	Viable counts of *Salmonella* Typhimurium (Log_{10} CFU/mL)																		
	(day) 0	Treatment			Withdrawal			Treatment			Withdrawal			Treatment			Withdrawal		
		1	4	7	8	11	14	15	18	21	22	25	28	29	32	35	36	39	42
0	4.60	4.54	4.87	3.78	4.57	3.96	4.72	3.59	4.40	3.87	4.40	4.67	4.46	3.68	4.99	4.33	3.28	4.15	4.35
0.1	4.29	3.72	3.97	4.39	4.50	3.55	3.17	3.50	3.85	3.34	4.83	4.13	4.65	3.68	4.15	3.23	3.87	4.56	4.12
4	4.48	0	0	0	4.56	4.40	2.76	4.07	3.62	4.02	3.45	4.19	3.20	2.67	3.50	3.12	3.73	3.88	3.79
100	4.14	0	0	0	0	0	0	0	0	0	0	0	0	0	0	0	0	0	0

Supplementary Table S2. Shedding levels (viable counts) of *Salmonella* Typhimurium CVCC541 in chickens exposed to different dosages of enrofloxacin. Chickens were challenged with *Salmonella* Typhimurium CVCC541 at their 4-day-old and treatment of enrofloxacin started at their 6 days old. Medication included three 7-day treatments alternated with 7-day withdrawal periods. Cloacal swabs were collected at their 5-day-old (day 0) and three times for each treatment period and withdrawal period. The values were means of five chickens of each group.

Dosage (mg/kg)	Variables	(day)	Treatment			Withdrawal			Treatment			Withdrawal			Treatment			Withdrawal		
		0	1	4	7	8	11	14	15	18	21	22	25	28	29	32	35	36	39	42
0	Total counts (Log CFU/mL)	4.86	4.96	5.46	3.67	4.20	4.42	4.52	3.56	4.45	4.44	4.50	5.14	4.34	4.47	4.73	5.18	4.45	4.24	4.61
	Percentage of less susceptible isolates (%)	0	0	0	0	0	0	0	0	0	0	0	0	0	0	0	0	0	0	0
	Percentage of non-susceptible isolates (%)	0	0	0	0	0	0	0	0	0	0	0	0	0	0	0	0	0	0	0
	Percentage of resistant isolates (%)	0	0	0	0	0	0	0	0	0	0	0	0	0	0	0	0	0	0	0
0.1	Total counts (Log CFU/mL)	4.77	3.47	4.49	4.36	4.29	4.83	4.33	4.37	4.35	4.05	4.12	4.56	4.23	4.35	4.64	4.12	4.65	4.88	4.88
	Percentage of less susceptible isolates (%)	0	0	0	0	0	0	2.42	4.91	1.32	4.56	16.2	17.5	6.49	1.68	8.12	7.11	10.98	0.69	1.20
	Percentage of non-susceptible isolates (%)	0	0	0	0	0	0	0	0	0	0	0	0	0	0	0	0	0	0	0
	Percentage of resistant isolates (%)	0	0	0	0	0	0	0	0	0	0	0	0	0	0	0	0	0	0	0
4	Total counts (Log CFU/mL)	4.69	0	0	2.82	4.27	3.83	3.10	4.18	3.74	4.08	4.31	4.12	3.62	3.42	4.29	2.79	4.44	4.52	5.47
	Percentage of less susceptible isolates (%)	0	0	0	0	0	0	32.3	0	0	23.9	23.1	66.1	18.7	52.3	30.2	60.3	36.4	27.7	15.1
	Percentage of non-susceptible isolates (%)	0	0	0	0	0	0	0	0	0	4.81	11.4	4.18	17.8	11.8	6.10	12.2	13.8	12.5	10.1
	Percentage of resistant isolates (%)	0	0	0	0	0	0	0	0	0	0	0	0	0	0	0	0	0	9.53	5.03
100	Total counts (Log CFU/mL)	4.82	0	0	0	0	3.68	4.32	0	0	0	0	3.23	4.26	4.09	4.31	5.04	5.12	5.22	5.73
	Percentage of less susceptible isolates (%)	0	0	0	0	0	0	0	0	0	0	0	59.3	66.4	95.4	87.2	67.9	66.9	69.1	81.7
	Percentage of non-susceptible isolates (%)	0	0	0	0	0	0	0	0	0	0	0	0	30.5	86.2	43.1	26.5	58.4	55.4	62.7
	Percentage of resistant isolates (%)	0	0	0	0	0	0	0	0	0	0	0	0	10.2	60.8	20.7	4.00	36.1	42.6	43.7

Supplementary Table S3. Total counts of coliforms and percentages of coliforms with different levels of reduced susceptibility in four treatment groups. Total counts, calculated from the coliforms grown on MacConkey plates without enrofloxacin; less susceptible isolates, grown on MacConkey supplemented with 0.125 mg/L of enrofloxacin; non-susceptible isolates, grown on MacConkey supplemented with 0.25

mg/L of enrofloxacin; resistant isolates, grown on MacConkey supplemented with 2 mg/L of enrofloxacin. The values were means of five chickens of each group.
